# Molecular Detection of *Rickettsia hoogstraalii* in *Hyalomma anatolicum* and *Haemaphysalis sulcata*: Updated Knowledge on the Epidemiology of Tick-Borne *Rickettsia hoogstraalii*

**DOI:** 10.3390/vetsci10100605

**Published:** 2023-10-04

**Authors:** Aneela Aneela, Mashal M. Almutairi, Abdulaziz Alouffi, Haroon Ahmed, Tetsuya Tanaka, Itabajara da Silva Vaz, Shun-Chung Chang, Chien-Chin Chen, Abid Ali

**Affiliations:** 1 Department of Zoology, Abdul Wali Khan University Mardan, Mardan 23200, Pakistan; 2 Department of Pharmacology and Toxicology, College of Pharmacy, King Saud University, Riyadh 11451, Saudi Arabia; mmalmutairi@ksu.edu.sa; 3 King Abdulaziz City for Science and Technology, Riyadh 12354, Saudi Arabia; asn1950r@gmail.com; 4 Department of Biosciences, COMSATS University Islamabad (CUI), Park Road, Chak Shahzad, Islamabad 45550, Pakistan; haroonahmed@comsats.edu.pk; 5 Laboratory of Infectious Diseases, Joint Faculty of Veterinary Medicine, Kagoshima University, Kagoshima 890-0065, Japan; k6199431@kadai.jp; 6 Centro de Biotecnologia and Faculdade de Veterinária, Universidade Federal do Rio Grande do Sul, Porto Alegre 90050-170, RS, Brazil; itabajara.vaz@ufrgs.br; 7 Department of Emergency Medicine, Ditmanson Medical Foundation Chia-Yi Christian Hospital, Chiayi 60002, Taiwan; 8 Department of Pathology, Ditmanson Medical Foundation Chia-Yi Christian Hospital, Chiayi 60002, Taiwan; hlmarkc@gmail.com; 9 Department of Cosmetic Science, Chia Nan University of Pharmacy and Science, Tainan 71710, Taiwan; 10 Ph.D. Program in Translational Medicine, Rong Hsing Research Center for Translational Medicine, National Chung Hsing University, Taichung 40227, Taiwan; 11 Department of Biotechnology and Bioindustry Sciences, College of Bioscience and Biotechnology, National Cheng Kung University, Tainan 70101, Taiwan

**Keywords:** ticks, Ixodidae, *Rickettsia hoogstraalii*, Pakistan

## Abstract

**Simple Summary:**

Ticks are hematophagous ectoparasites that spread diseases to both animals and humans through their bites. They are notorious for carrying various disease-causing agents, such as viruses, protozoa, and bacteria, which present substantial risks to both human and animal well-being. Continuous changes in the climate can impact both tick distribution and abundance. Understanding of the epidemiology of tick-borne *Rickettsia hoogstraalii* is limited, with gaps in its molecular detection, genetic characterization, and absence of data, especially from Pakistan. This study aimed to use molecular methods to genetically analyze *Rickettsia* species, particularly *R. hoogstraalii*, in Pakistan while also contributing new insights into the pathogen′s global epidemiology. For this purpose, ticks were collected from different hosts, including goats, sheep, and cattle, from six districts of Khyber-Pakhtunkhwa, Pakistan. This study is the first to genetically characterize *R. hoogstraalii* in *Hyalomma anatolicum* ticks globally and *Haemaphysalis sulcata* in Pakistan. This species was first described in 2006 in Croatia and has also been detected in different species of ticks in different countries. The pathogenicity of *R. hoogstraalii* in vertebrate hosts is not well understood. Encouraging additional research is essential to unveil the involvement of ticks in the transmission and persistence of *R. hoogstraalii* across various host species.

**Abstract:**

Ticks are hematophagous ectoparasites that transmit pathogens to animals and humans. Updated knowledge regarding the global epidemiology of tick-borne *Rickettsia hoogstraalii* is dispersed, and its molecular detection and genetic characterization are missing in Pakistan. The current study objectives were to molecularly detect and genetically characterize *Rickettsia* species, especially *R. hoogstraalii,* in hard ticks infesting livestock in Pakistan, and to provide updated knowledge regarding their global epidemiology. Ticks were collected from livestock, including goats, sheep, and cattle, in six districts of Khyber Pakhtunkhwa (KP) Pakistan. Overall, 183 hosts were examined, of which 134 (73.2%), including goats (number = 39/54, 72.2%), sheep (23/40, 57.5%), and cattle (71/89, 80%) were infested by 823 ticks. The most prevalent tick species was *Rhipicephalus microplus* (number = 283, 34.3%), followed by *Hyalomma anatolicum* (223, 27.0%), *Rhipicephalus turanicus* (122, 14.8%), *Haemaphysalis sulcata* (104, 12.6%), *Haemaphysalis montgomeryi* (66, 8.0%), and *Haemaphysalis bispinosa* (25, 3.03%). A subset of 210 ticks was selected and screened for *Rickettsia* spp. using PCR-based amplification and subsequent sequencing of rickettsial *gltA* and *ompB* fragments. The overall occurrence rate of *R. hoogstraalii* was 4.3% (number = 9/210). The DNA of *Rickettsia* was detected in *Hy. anatolicum* (3/35, 8.5%) and *Ha. sulcata* (6/49, 12.2%). However, no rickettsial DNA was detected in *Rh. microplus* (35), *Rh. turanicus* (35), *Ha. montgomeryi* (42), and *Ha. bispinosa* (14). The *gltA* and *ompB* fragments showed 99–100% identity with *R. hoogstraalii* and clustered phylogenetically with the corresponding species from Pakistan, Italy, Georgia, and China. *R. hoogstraalii* was genetically characterized for the first time in Pakistan and *Hy. anatolicum* globally. Further studies should be encouraged to determine the role of ticks in the maintenance and transmission of *R. hoogstraalii* in different hosts.

## 1. Introduction

Ticks are obligate blood-sucking ectoparasites distributed all over the world, especially in tropical and subtropical areas [1,2,3]. The most important hard ticks that transmit pathogens and affect domestic and wild animals belong to different genera such as *Rhipicephalus*, *Hyalomma*, *Haemaphysalis*, *Ambylomma*, and *Ixodes* [4]. These hematophagous ectoparasites play a significant role in transmitting pathogens, encompassing bacteria, protozoans, and viruses that lead to zoonotic outcomes threatening human and animal health [5,6]

*Rickettsia* species are obligatory intracellular Gram-negative bacteria that are divided into major groups: the spotted fever group (SFG), the typhus group, the bellii group, and the limioniae group [7,8]. Among these, tick-borne SFG *Rickettsia* spp. include a large number of zoonotic agents and are considered important pathogens causing SFG [5,9,10]. *Rickettsia* spp. of the SFG are mostly transmitted by hard ticks (Ixodidae) to vertebrate hosts [5]. In addition, the human pathogenicity of several rickettsial species has been described, and rickettsial species with undetermined pathogenicity have been observed in ticks [5,11,12]. 

*Rickettsia hoogstraalii* is a member of the SFG with unknown pathogenicity and is closely related to *Rickettsia felis*, an emerging pathogen known to be spread through arthropods, especially ticks and fleas [13,14,15,16]. *Rickettsia hoogstraalii* was first reported in 2006 in *Ha. sulcata* ticks in Croatia [17] and later on was detected in various tick species of the genera *Heamaphysalis, Rhipicephalus*, *Argas*, *Dermacentor*, *Carios*, *Ixodes*, and *Africaniella* in Croatia, Pakistan, Georgia, Spain, Cyprus, India, Ethiopia Turkey, Italy, Greece, Iran, USA, Namibia, Zambia, Romania, China, Africa, and Anatolia [15,16,17,18,19,20,21,22,23,24,25,26,27,28,29,30,31,32,33,34,35,36,37,38,39,40,41,42]. Notably, it has been detected in *Ha. montgomery,* infesting goat and sheep from Pakistan [15].

Pakistan is an agricultural country, and livestock is an important part of its economy, as different animals are important sources of income [Pakistan Economic Survey 2022–2023] [43]. Ticks of different genera, such as *Hyalomma*, *Heamaphysalis*, *Rhiphcephalus*, *Amblyomma*, *Ixodes*, *Ornithodoros*, *Argas*, *Carios*, and *Nosomma,* have been reported infesting livestock and wild animals in Pakistan [8,44,45,46,47,48,49,50,51]. These ticks are capable of transmitting pathogens, including *Rickettsia* spp., *Theileria* spp., *Babesia* spp., and *Anaplasma* spp. [44,45,46]. In Pakistan, studies have reported that tick-borne pathogens infect domestic and wild animals [8,44,45,49,51,52,53,54]. There is no available information regarding the genetic characterization of tick-borne *R. hoogstraalii* in Pakistan, and knowledge of its global epidemiology is limited. To address this gap, this study aimed to detect *Rickettsia* species, especially *R. hoogstraalii*, in ticks infecting livestock hosts in Pakistan and to update and summarize dispersed information on its global epidemiology.

## 2. Materials and Methods 

### 2.1. Study Area

**Figure 1:**
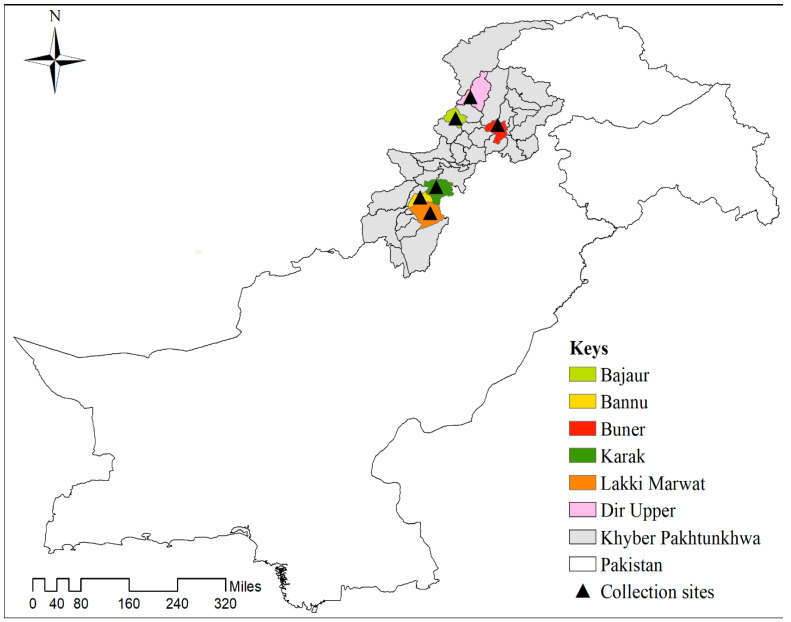
Map showing the locations (black triangles) of tick collection in specific districts of Khyber Pakhtunkhwa (KP), Pakistan.

Present study was conducted in six districts of Khyber Pakhtunkhwa: Buner (34°33′43.6″ N 72°24′37.1″ E), Lakki Marwat (32°36′53.5″ N 70°54′37.6″ E), Bannu (32°59′14.3″ N 70°39′33.0″ E), Karak (33°11′53.7″ N 71°07′57.2″ E), Bajaur (34°42′09.5″ N 71°37′58.6″ E), and Dir-Upper (35°11′52.7″ N 71°52′46.1″ E). Geographic coordinate data were collected using a global positioning system (GPS) and stored in Microsoft Excel v. 2016 (Microsoft Corp., Redmond, WA, USA) for processing. The study area map (Figure 1) was drawn in ArcGIS v. 10.3.1 (ESRI, Redlands, CA, USA).

### 2.2. Ticks Collection and Identification 

Ticks were collected between June 2021 and August 2022 from asymptomatic livestock hosts (cattle, goats, and sheep) at different sites. Ticks were collected from the bodies of the hosts, regardless of their particular location within the planned survey zones or times, whenever they were found in different farms, open fields, or free-roaming animals in pastures. With the use of forceps, 1–10 ticks per animal were collected from each host while examining their entire body. The collected specimens were washed with distilled water followed by 70% ethanol to remove contaminants and stored in Eppendorf tubes with 99.98% ethanol. Morphological identification was performed using a StereoZoom microscope (HT StereoZoom), following taxonomic keys [55,56] and stored in 2 mL microtubes for further molecular analysis.

### 2.3. DNA Extraction and PCR

Location- and gender-wise, a total of 210 (79 females, 24 males, 107 nymphs) ticks were randomly selected as representatives of the collected ticks for DNA extraction and PCR, comprising 49 specimens (19 F, 4 M, 26 N) from Bajaur, 42 (17 F, 5 M, 20 N) from Buner, 35 (14 F, 6 M, 15 N) from Dir-Upper, 28 (9 F, 2 M, 17 N) from Bannu, 18 (10 F, 3 M, 5 N) from Lakki-Marwat, and 28 (10 F, 4 M, 14 N) from Karak districts. Tick specimens were rinsed with PBS and distilled water followed by 70% ethanol. The washed specimens were kept in an incubator for 20–30 min at 37 °C. Each specimen was cut separately using a sterile blade, and DNA was extracted using the phenol-chloroform method [57]. DNA was quantified using a Nanodrop spectrophotometer (Nano-Q, Optizen, Daejeon, South Korea). The extracted DNA was tested for the presence of *Rickettsia* spp. using PCR targeting *gltA*, *ompA*, and *ompB* fragments. The PCR reaction mixture was carried out in a total volume of 25 µL, consisting of 1 µL of each (forward and reverse) primer (10 µM) (Table 1), 2 µL genomic DNA template (100 ng/µL), 12.5 µL Master mix (2×) (Thermo Fisher Scientific, Inc., Waltham, MA, USA), and 8.5 µL PCR water. *Rickettsia massilliae* DNA was utilized as a positive control while “nuclease free” water was used as a negative control. The amplified PCR products were electrophoresed on a 2% gel, stained with ethidium bromide, and visualized using GelDoc (BioDoc-It™ Imaging Systems; Upland, CA, USA). The DNA purification Kit (Invitrogen™JetFlex™, Invitrogen, Waltham, MA, USA) was used to purify amplicons prior to the sequencing process in both directions by MACROGEN (Seoul, Republic of Korea).

### 2.4. Sequencing and Phylogenetic Analysis

The obtained sequences were trimmed using Seqman 5.0 (DNASTAR, Inc., Madison, WI, USA) to remove poor sequencing reads and primer contaminations. All the obtained sequences were identical; hence, a single consensus sequence was obtained. The consensus sequences were submitted to the Basic Local Alignment Search Tool (BLASTn; National Center for Biotechnology Information [NCBI)]). Higher-identity sequences were aligned using BioEdit alignment editor v. 7.0.5 [61] and were subjected to ClustalW Multiple alignment [62]. The individual phylogenetic trees of *gltA* and *ompB* were constructed in accordance with the maximum likelihood method in Molecular Evolutionary Genetics Analysis (MEGA-XI) software [63], using the MUSCLE algorithm [64]. A similar outcome was observed for all the available methods. However, due to its ability to evaluate different phylogenetic trees and models under a statistical framework, the maximum likelihood method is recommended as the actual method for the best evolutionary analysis [65]. Statistical analysis of the nodes was performed using bootstrap resampling analysis, which involved 1000 replicates. This approach provided a rigorous assessment of the reliability of the tree branching patterns and relationships [63]. The acquired fragments of *gltA* and *ompB* were used to determine their final positions in the dataset.

### 2.5. Literature Search

A literature search was carried out using databases (Google Scholar, Web of Science, PubMed and ScienceDirect) to collect published studies on the detection of *R. hoogstraalii* in various ticks, animals, humans, or soils. The search keywords used included but were not limited to ticks, tick-borne diseases, domestic animals, small ruminants, livestock, sheep, goat, zoonosis, and *R. hoogstraalii*. A varied keyword approach was employed to gather full-text research articles, reviews, short communications, and conference papers from different sources. In order to identify relevant articles, the reference lists of the retrieved articles were also examined (Table 2).

## 3. Results

### 3.1. Ticks and Host Description

Among 183 examined livestock hosts in Buner district (12 goats, 10 sheep, and 14 cattle), Bajaur (10 goats, 8 sheep and 15 cattle), Lakki Marwat (12 sheep and 14 cattle), Bannu (8 goats and 15 cattles), Dir-Upper (8 goats, 10 sheep and 10 cattle), and Karak (16 goats and 21 cattle), 134 were tick-infested (Table 3). Nymphs were most prevalent (353, 42.8%), while the least prevalence was observed for females (297, 36%) and males (173, 21%) (Table 3). A total of 823 ticks from different life stages were collected and morphologically classified into three genera and five species as follows: *Rhipicephalus* spp. (405 specimens, 49.2% of all ticks), *Hyalomma* spp. (223 specimens, 27% of all ticks), and *Haemaphysalis* spp. (195 specimens, 23% of all ticks). The most abundant species was *Rh.* (*Boophilus*) *microplus* (283 specimens, 34.3% of all ticks), followed by *Hy. anatolicum* (223 specimens, 27% of all ticks), *Rh. turanicus* (122 specimens, 14.8% of all ticks), *Ha. sulcata* (104 specimens, 12.6% of all ticks), *Ha. montgomeryi* (66 specimens, 8%), and *Ha. bispinosa* (25 specimens, 3.03% of all ticks). The overall prevalence of tick infestation among livestock hosts was 73.2%, with the heaviest tick burden recorded in domestic animals of district Bajaur (158, 19.9%), followed by Buner (146, 17.7%), Karak (144, 17.4%), Lakki Marwat (133, 16.1%), Bannu (126, 15.3), and Dir-Upper (116, 14%). Among domestic animals, cattle were infested the most with 506 ticks, including *Rh. microplus* (283) and *Hy. anatolicum* (223), followed by goats, infested with 202 ticks, including *Rh. turanicus* (122), *Ha. sulcata* (49) and *Ha. montgomeryi* (31). Sheep were the least infested with 115 ticks, including *Ha. sulcata* (55), *Ha. montgomeryi* (35), and *Ha. bispinosa* (25).

### 3.2. Detection of Rickettsial DNA in Ticks

Ticks positive for rickettsial gltA were also positive for the *ompB* fragment, whereas *ompA*-based PCR amplification was unsuccessful in all PCR reactions. The overall occurrence of *Rickettsia* spp. was 4.3% (9/210) based on *gltA* and *ompB* partial fragments. The occurrence of *Rickettsia* spp. was highest in *Ha. sulcata* (6/49, 12.2%) followed by *Hy. anatolicum* (3/35, 8.5%). However, no rickettsial DNA was detected in *Rh. microplus* (35), *Rh. turanicus* (35), *Ha. montgomeryi* (42), and *Ha. bispinosa* (14). Location-wise, the occurrence of Rickettsia-positive ticks was highest in district Bannu (2/28, 7.1%), followed by Bajaur (3/49, 6.1%), Lakki Marwat (1/18, 5.5%), Buner (2/42, 4.7%), and Dir-Upper (1/35, 2.8%) (Table 3).

### 3.3. Sequences and Phylogenetic Analysis

**Figure 2:**
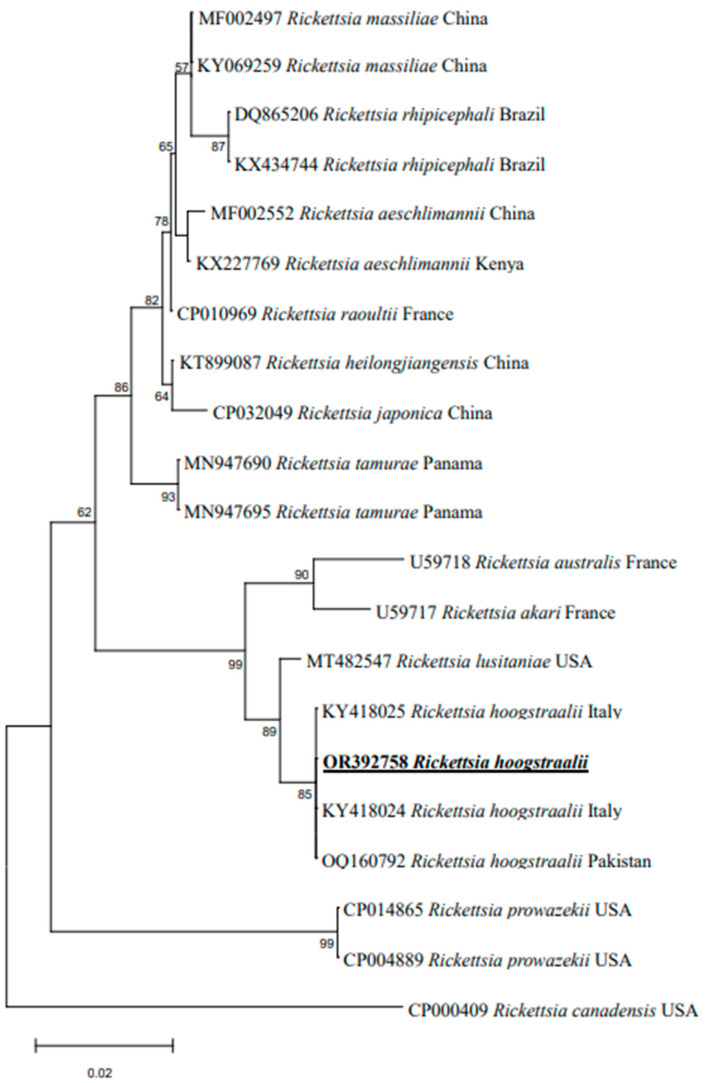
A maximum likelihood phylogenetic tree of *R. hoogstraalii* was constructed based on the gltA fragment. R. canadensis was used as an outgroup. The bootstrap values (1000-replication) are shown at each node. The obtained sequence (OR392758) of the present study is marked in bold and underlined font.

**Figure 3:**
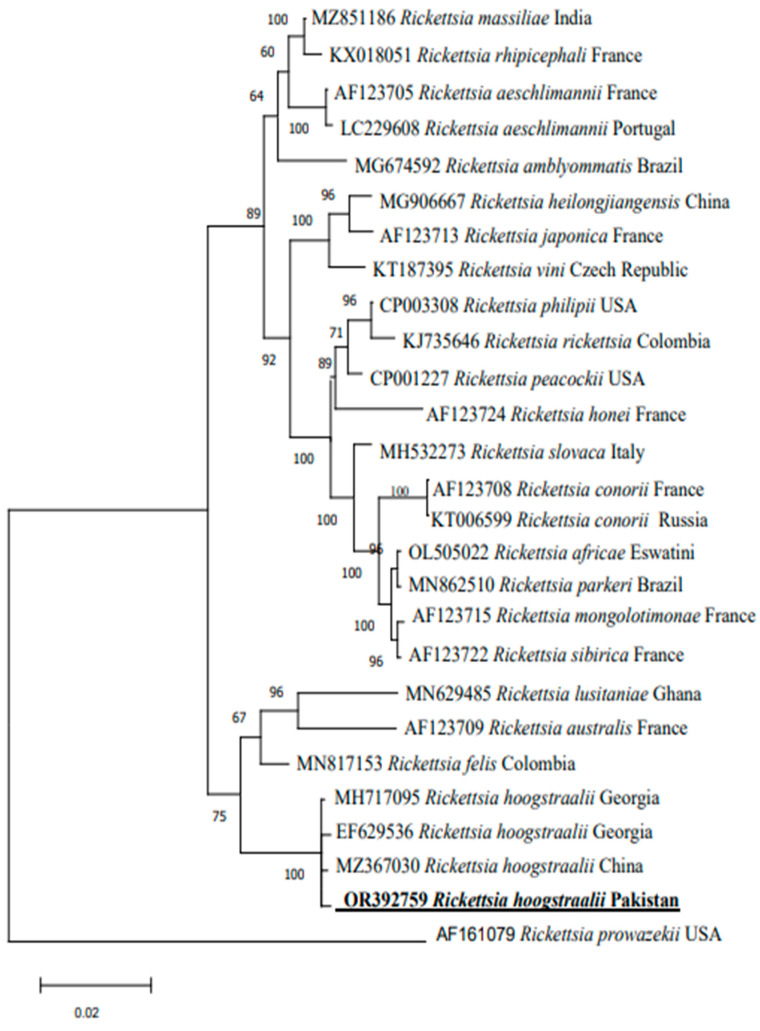
A maximum likelihood phylogenetic tree of *R. hoogstraalii* was constructed based on the *ompB* partial fragment. *R. prowazekii* was used as the outgroup. Bootstrap values (1000-replication) are shown at each node. The obtained sequence (OR392759) of the present study is marked in bold and underlined font.

After a BLAST search of the NCBI database, the *gltA* sequence revealed 100% identity and 100% query identity with *R. hoogstraalii* reported in Italy and Pakistan. On the other hand, the ompB (773 bp) sequence of *R. hoogstraalii* revealed 99.2–99.7% high identity and 100% query to the reported sequences from China and the USA. In the phylogenetic tree of gltA, the obtained sequences clustered with those of *R. hoogstraalii* from Italy (KY418024 and KY418025) and Pakistan (OQ160792) (Figure 2). In the phylogenetic tree of *ompB*, the obtained sequence clustered with *R. hoogstraalii* from Georgia (EF629536 and MH717095) and China (MZ367030) (Figure 3).

## 4. Discussion

Ticks cause economic losses to the livestock industry and transmit various pathogens, including SFG *Rickettsia* spp., to humans and wild and domestic animals. There is a huge variety of *Rickettsia* spp., of which few have been proven to be zoonotic [5]. *Rickettsia hoogstraalii* is a member of the SFG *Rickettsia,* but there are no available reports on its pathogenicity in vertebrates [33]. The diagnosis of *Rickettsia* in ticks, not only for the identification of infected ticks but also for the assessment of exposure risk to humans, is important [66,67]. Previously, various studies have documented the occurrence of diverse *Rickettsia* spp. in various ticks infesting different hosts in Pakistan, but there is a lack of information regarding the occurrence and genetic characterization of *R. hoogstraalii*. To fill this gap, we detected and genetically characterized *R. hoogstraalii* in hard ticks infesting livestock. The collected ticks were taxonomically identified as *Rh. microplus*, *Rh. turanicus*, *Hy. anatolicum*, *Ha. bispinosa*, and *Ha. sulcata* and screened for the detection of rickettsial DNA. Among these, *R. hoogstraalii* was identified in *Hy. anatolicum* and *Ha. sulcata* based on *gltA* and *ompB* sequences for the first time in Pakistan.

Tick species such as *Rh. microplus, Rh. turanicus, Rh. sanguienus, Rh. haemaphysaloides, Hy. anatolicum, Hy. dromedarii, Ha. sulcata, Ha. bispinosa, Ha. kashmirensis, Ha. cornupunctata,* and *Ha. montgomeryi* have been found to infect different livestock hosts (especially cattle, goats, and sheep) in different regions of Pakistan [2,8,15,44,48,49,50]. *Rhipicephalus microplus* and *Hy. anatolicum*, which are the most prevalent in the area, were found most frequently [2,8]. The environmental conditions in the different survey districts varied from one another. The annual mean temperature of study areas such as Lakki Marwat, Bannu, Bajaur, Upper-Dir, Karak, and Buner were 30–42 °C and 4–17 °C, recorded in the summer and winter, respectively, (worldweatheronline.com: accessed on 1 March 2023). High summer temperatures in the target area were correlated with increased tick infestation compared to winter; therefore, high tick infestation was noted during summer. Moreover, several tick species have been found to exhibit low incidences of infestation as a result of lower temperatures in certain districts. These results are consistent with previous regional reports [2,68,69].

*Hyalomma anatolicum* and *Ha. sulcata* infesting cattle, goats, and sheep were found positive for *R. hoogstraalii*. Previously, *R. hoogstraalii* has been detected in various tick genera, including *Haemaphysalis*, *Rhipicephalus*, *Argas*, *Dermacentor*, *Carios*, *Ixodes*, and *Africaniella,* infesting goats, sheep, cattle, cows, mouflons, lizards, bat, dog, and birds in different countries [15,16,17,18,19,20,21,22,23,24,25,26,27,28,29,30,31,32,33,34,35,36,37,38,39,40,41,42]. Recently, *R. hoogstraalii* was reported to contain a short fragment of *gltA* in *Ha. montgomeryi* infesting goats and sheep in Pakistan [15]. *Rickettsia hoogstraalii* has been detected in all life stages of different ticks, such as adult females, males, larvae, and nymphs [15,28,33,34]. So far, information about the detection of *R. hoogstraalii* in *Hy. anatolicum* and *Ha. sulcata* ticks infesting livestock hosts such as cattle, goats, and sheep were unavailable. Herein, *R. hoogstraalii* was detected for the first time in *Hy. anatolicum* globally and in *Ha. sulcata* in Pakistan. This study presents the first molecular evidence of *R. hoogstraalii* in nymphs and adult female ticks of *Hy. anatolicum* and *Ha. sulcata*. It also suggests that these ticks may play a possible role in the spread of *R. hoogstraalii*. *R. hoogstraalii* DNA was detected both in nymph and adult female ticks. Consequently, there are chances that this *Rickettsia* was ingested from the blood of the infected hosts. *Rickettsia* spp.-infected ticks may pose unknown health risks to livestock owners, indicating that other tick species in the area might be potential vectors of these infectious agents [5].

Genetic markers such as *gltA*, *ompA,* and *ompB* have been used to distinguish several *Rickettsia* spp. at the species level [58,60,70]. Thus, the characterization of *R. hoogstraalii* has been validated through the use of these standard markers [15,16,20]. We molecularly detected *R. hoogstraalii* based on the *gltA* and *ompB* sequences. Using these genetic markers, the obtained sequences were closely related to *R. hoogstraalii* in the Palearctic and Neotropic regions. Additionally, sequence analysis of *gltA* and *ompB* showed that *R. hoogstraalii* is closely related to *R. felis*, making it a distinct species in the spotted fever group [48]. PCR-based detection of this species was also attempted based on the *ompA* fragment; however, the amplification was unsuccessful, as reported previously [28]. Amplification failure is common for *ompA,* which may be the absence of targeted genes, as demonstrated by the rickettsial transition group, or due to primer mismatch [35,71]. There is no available information regarding the pathogenicity of *R. hoogstraalii* in vertebrate hosts including humans [33], and its zoonotic outcomes are yet to be determined in Pakistan. Further studies are required to elucidate the pathogenicity of *R. hoogstraalii* in mammals.

## 5. Conclusions

This study provides preliminary information regarding the occurrence of *R. hoogstraalii* in *Hyalomma* and *Haemaphysali*s ticks including *Hy. anatolicum* and *Ha. sulcata*. To our knowledge, tick-borne *R. hoogstraalii* was detected and genetically characterized for the first time in globally in *Hy. anatolicum* and for the first time in Pakistan in *Ha. sulcata*. These findings indicate that ticks that infest goats, sheep, and cattle ultimately pose unknown health risks to livestock holders who mostly share living habitats. These findings enhance our understanding of the occurrence of *R. hoogstraalii* in ticks parasitizing livestock in Pakistan. To prevent zoonotic outcomes, it is important to examine the vector potential of different ticks for other rickettsial pathogens.

## Figures and Tables

**Table 1:** List of primers used in the present study for the amplification of ***Rickettsia*** spp.

Gene	Primer	Primers Sequence 5′-3′	Amplicons	Reference
* Rickettsia gltA *	CS-78	GCAAGTATCGGTGAGGATGTAAT	401 bp	[58]
	CS-323	GCTTCCTTAAAATTCAATAAATCAGGAT		
* Rickettsia ompA *	Rrl9O.70	ATGGCGAATATTTCTCCAAAA	631 bp	[59]
	Rr190.701n	GTTCCGTTAATGGCAGCATCT		
* Rickettsia ompB *	120-M59	CCGCAGGGTTGGTAACTGC	862 bp	[60]
	120–807	CCTTTTAGATTACCGCCTAA		

**Table 2:** Global epidemiology of tick-borne *R. hoogstraalii* detected in different ticks infesting various hosts using different methods.

Country/Year	Tick Specie/Source	Detected in Host	Serologically/Molecularly (PCR)	Reference
Croatia 2006	*Ha. sulcata *	Sheep	PCR	[17]
		Goats		
Georgia, USA 2007 “*Candidatus R. hoogstraalii*”	*Carios capensis*	Brown pelicans	PCR	[18]
Southeastern Spain 2008	* Ha. punctata *	Vegetation	PCR	[19]
	* Ha. sulcata *			
La Rioja, Spain 2008	*Ha. punctata*	Sheep	PCR	[20]
	*Ha. sulcata*	Cow		
Western India 2011–2012	* C. capensis *	Seabird	PCR	[21]
Croatia 2010	*Ha. sulcata*	Sheep	PCR/TEM	[22]
Cyprus 2011	* Ha. punctata *	Mouflons (Wild sheep)	PCR	[23]
Ethiopia 2012	*Ar. persicus*	Cracks and crevices of livestock areas	PCR	[24]
Turkey 2014	*Ha. parva*	Humans	PCR	[25]
Turkey 2016	*Ha. parva*	Humans	PCR	[26]
Italy 2016	*Ha. punctata*	Mouflons	PCR	[27]
	*Ha. sulcata*			
Italy 2017	* I. ricinus *	Lizards	PCR	[28]
	* Ha. sulcata *			
Greece 2017	* Ha. parva *	Dog	PCR	[29]
Greece 2019	* Ha. sulcata *	Goats	PCR	[30]
	* Ha. parva, * and *Ha. sulcata*	Sheep		
Iran 2020	*Ar. persicus*	Aviary	PCR	[31]
DESERT SOUTHWEST, USA 2020	*Ar. persicus*	Birds	PCR	[32]
Turkey 2020	* Ha. parva *	Wild animals include wolves, fox, hare, and lynx,	PCR	[33]
Georgia 2020	*D. marginatus*	Domestic animals	PCR	[34]
	*Ha. sulcata*			
Namibia 2020	*Argus transgariepinus*	Bat	PCR	[35]
Zambia 2021	* Ar. walkerae *	Chicken coop	PCR	[36]
Italy 2021	*I. ricinus*	Domestic animals	PCR	[37]
Romania 2022	* Rh. rossicus *	Dog	PCR	[38]
China 2022	* Ha. montgomeryi *	Goats	PCR	[39]
China 2022	*Ar. persicus*	From Cracks in hen house	PCR	[40]
Africa 2022	* Africaniella transversale *	Python regius	PCR	[41]
Anatolia 2022	*Ha. parva*	Cattle, Sheep, and Goats	PCR	[42]
	*Ha. sulcata*			
Pakistan 2023	* Ha. montgomeryi *	Goats	PCR	[15]
		Sheep		
Spain 2023	*Ha. formosensis*	Vegetation	PCR	[16]

**Table 3:** Information regarding ticks, hosts, locality, and the molecular detection of *R. hoogstraalii* in this study.

District	Examined Host	Ticks Species	Infested/Examined (%)	Number of Ticks (%) (F, M, N)	Ticks Subjected to PCR (F, M, N)	*Rickettsia hoogstraalii* Detected via Both *gltA,* and *ompB*
Buner	Goats	*Rh. turanicus*	8/12 (66.6)	25 (17.1), (7,5,13)	2, 1, 4	-
		*Ha. sulcata*		18 (11.3), (8,3,7)	2, 2, 3	1 N
	Sheep	*Ha. bispinosa*	6/10 (60)	15 (10.2), (7,3,5)	4, 0, 3	-
		*Ha. sulcata*		12 (7.6), (4,3,5)	2, 1, 4	1 N
		*Ha. montgomeryi*		10 (6.8), (3,2,5)	3, 1, 3	-
	Cattle	*Rh. microplus*	10/14 (71.4)	66 (41.7), (21,14,31)	4, 0, 3	-
Total			24/36 (66.6)	146 (17.7), (50, 30, 66)	17, 5, 20	2 N
Bajaur	Goats	*Rh. turanicus*	6/10 (60)	18 (11.3), (5,4,9)	3, 0, 4	-
		*Ha. sulcata*		13 (8.2), (3,2,8)	3, 1, 3	1 N
		*Ha. montgomeryi*		9 (5.6), (3,2,4)	3, 1, 3	-
	Sheep	*Ha. bispinosa*	5/8 (62.5)	10 (6.5), (4,2,4)	2, 1, 4	-
		*Ha. sulcata*		15 (9.3), (5,2,8)	4, 0, 3	2F
	Cattle	*Rh. microplus*	13/15(86.6)	50 (31.6), (19,10,21)	2, 0, 5	-
		*Hy. anatolicum*		43 (26.0), (16,9,18)	2, 1, 4	-
Total			25/33 (75.7)	158 (19.9), (55, 31, 72)	19, 4, 26	2 F, 1 N
Lakki Marwat	Sheep	*Ha. sulcata*	6/12 (50)	18 (13.5), (5,4,9)	3, 1, 3	-
		*Ha. montgomeryi*		13(9.7), (5,2,6)	2, 1, 4	-
	Cattle	*Rh. microplus*	12/14 (85.7)	60 (45.1), (20,17,23)	2, 1, 4	-
		*Hy. anatolicum*		42 (13.5), (15,10,17)	3, 0, 4	1 N
Total			18/26 (75)	133 (16.1), (45, 33, 55)	10, 3, 5	1 N
Bannu	Goats	*Rh. turanicus*	5/8 (62.5)	19 (15.0), (7,4,8)	3, 1, 3	-
		*Ha. montgomeryi*		10 (7.9), (4,2,4)	3, 0, 4	-
	Cattle	*Rh. microplus*	12/15 (80)	47 (44.3), (19,8,20)	2, 0, 5	-
		*Hy. anatolicum*		50 (39.6), (18,11,21)	1, 1, 5	1F, 1 N
Total			17/23 (74)	126 (15.3), (48, 25, 53)	9, 2, 17	1 F, 1 N
Dir-Upper	Goats	*Rh. turanicus*	6/8 (75)	30 (25.8), (11,4,15)	2, 2, 3	-
		*Ha. sulcata*		18 (15.5), (6,2,10)	4, 1, 2	1 N
	Sheep	*Ha. sulcata*	6/10 (60)	10 (8.6), (4,2,4)	3, 1, 3	
		*Ha. montgomeryi*		12 (10.3), (4,3,5)	2, 2, 3	-
	Cattle	*Hy. anatolicum*	8/10 (80)	46 (27.7), (14,14,18)	3, 0, 4	-
Total			20/28 (71.4)	116 (14.1), (39, 25, 52)	14, 6, 15	1 N
Karak	Goats	*Rh. turanicus*	14/16 (87.5)	30 (20.8), (12,5,13)	2, 1, 4	-
		*Ha. montogomeryi*		12 (8.3), (4,2,6)	3, 1, 3	-
	Cattle	*Rh. microplus*	16/21 (76.1)	60 (41.6), (22,13,25)	3, 0, 4	-
		*Hy. anatolicun*		42 (23.2), (16,9,17)	2, 2, 3	-
Total			30/37 (81.0)	144 (17.5), (54, 29, 61)	10, 4, 14	-
Overall Total			134/183 (73.2)	823 (297, 173, 353)	210 (79, 24, 107)	9 (3 F, 6 N) (4.3%)

Note: N = nymphs; M = males; F = adult females.

## Data Availability

The data set of the current study can be found in the online repository under the accession numbers present in the article.
